# Clinical Outcome of Children With Antenatally Diagnosed Hydronephrosis

**DOI:** 10.3389/fped.2019.00103

**Published:** 2019-03-29

**Authors:** Benedetta Chiodini, Mehran Ghassemi, Karim Khelif, Khalid Ismaili

**Affiliations:** ^1^Department of Pediatric Nephrology, Hôpital des Enfants Reine-Fabiola, Université Libre de Bruxelles, Brussels, Belgium; ^2^Department of Medical Imaging, Hôpital des Enfants Reine-Fabiola, Université Libre de Bruxelles, Brussels, Belgium; ^3^Department of Pediatric Urology, Hôpital des Enfants Reine-Fabiola, Université Libre de Bruxelles, Brussels, Belgium

**Keywords:** CAKUT, antenatal, fetal hydr, reflux vesico-ureteric, urinary obstruction

## Abstract

Fetal renal pelvis dilation is a common condition, which is observed in 1–4. 5% of pregnancies. In many cases, this finding resolves spontaneously. However, sometimes it may be a signal of significant urinary tract pathologies. The main abnormalities found after birth are uretero-pelvic junction stenosis, primary vesicoureteral reflux, megaureter, duplex kidneys, and posterior urethral valves, with uretero-pelvic junction stenosis and primary vesicoureteral reflux accounting for most of the cases. Diagnosis, management, and prognosis at short and longer term of these conditions will be reviewed in this article.

## Introduction

Fetal renal pelvis dilation is a common condition, which is observed in 1–4.5% of pregnancies (1, 2). The dilation can concern either the renal pelvis alone (also referred to as pyelectasis) or the dilation of both the pelvis and the calices (also referred to as pelvicaliectasis or hydronephrosis). In practice, these terms are often used interchangeably to refer to a dilated renal collecting system regardless of its etiology (3). Correlation between prenatal and post-natal findings and the final urological diagnosis has been problematic, partly because of the lack of uniformity in defining and grading urinary tract dilation. Several grading systems have been used, such as the descriptive (mild-moderate-severe), the quantitative (antero-posterior renal pelvis diameter), or the semiquantitative (Society for Fetal Urology (SFU) grading system) (4).

During pregnancy, the third-trimester threshold value for the antero-posterior renal pelvis diameter of 7 mm is the most widely used criterion in order to select patients requiring post-natal investigation (3, 4). Fetal distension of the urinary collecting system may be simply a dynamic and physiologic process which resolves spontaneously in 36–80% of cases after birth (5–8). However, in some cases renal pelvis dilation can signal the presence of severe urinary tract pathologies (9), especially in patients with significant hydronephrosis (3).

Thanks to its safety, wide feasibility, excellent anatomical resolution, and low-cost, ultrasound (US) is the first examination to perform after birth (7).

In 2014, eight American societies with special interest in maternal-fetal medicine, urology, nephrology, and radiology, collaborated to provide a consensus on the terminology and a unified sonographic grading system for perinatal urinary tract (UT) dilation. The Consensus panel proposed a standardized scheme for follow-up evaluation depending on the severity of the UT dilation grade and other US findings. As regards the terminology, the panel recommends the consistent use of the term “UT dilation” (4).

**Figure 1:**
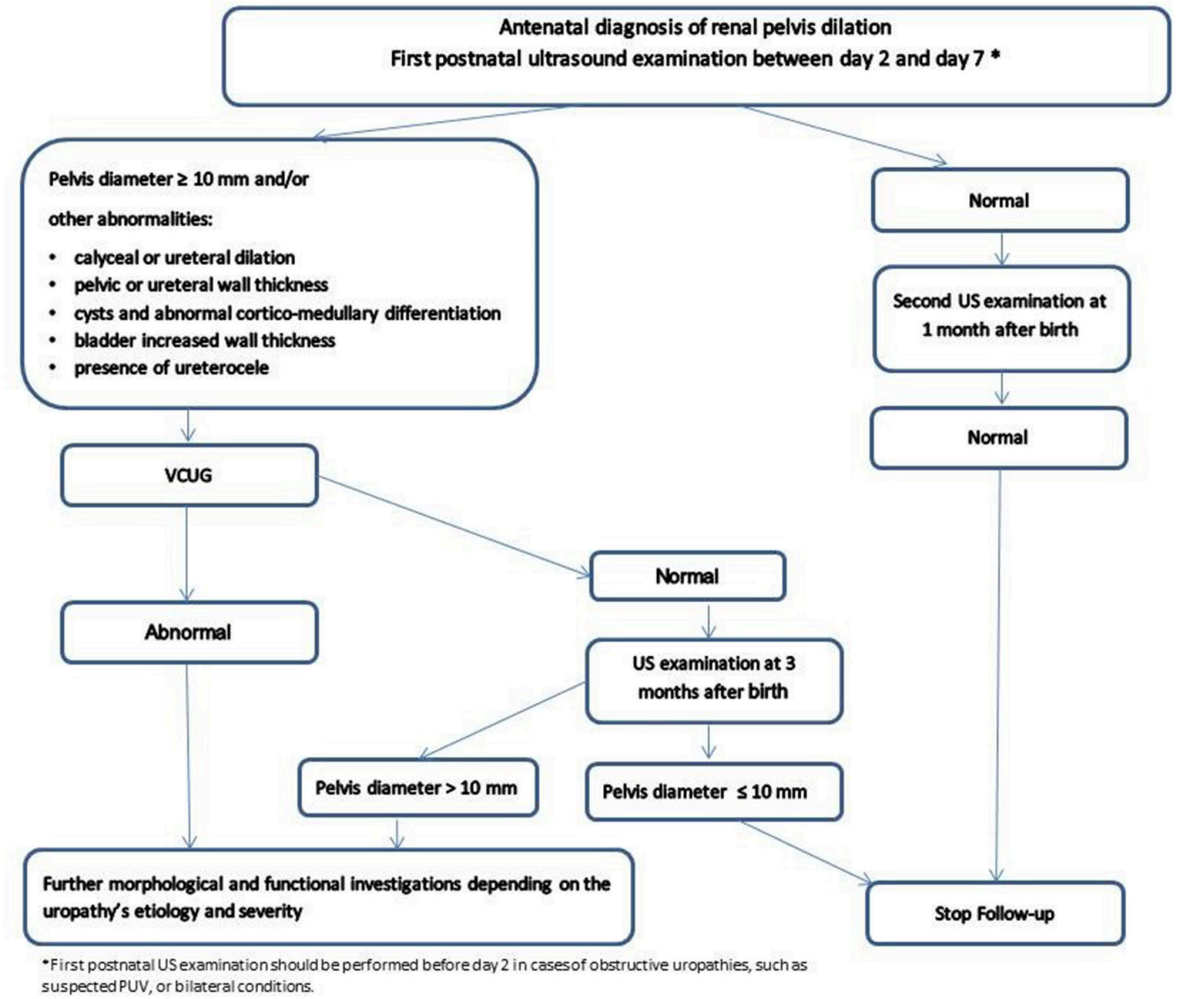
Antenatally detected urinary tract dilation and post-natal imaging strategy.

A post-natal antero-posterior renal pelvis diameter of 10 mm is the most commonly accepted upper limit threshold value for normality (10) while a pelvis diameter >15 mm is mostly associated with significant uronephropathies (4, 11). Based on our experience (3, 7, 11) and on the American (4) and European recommendations (12), we have proposed an algorithm for a rational post-natal imaging strategy (Figure 1).

In addition to the renal pelvis dilation, the need for post-natal investigations is also determined by the presence of other anomalies, such as a calyceal or ureteral dilatation, pelvic or ureteral wall thickening, cysts and abnormal cortico-medullary differentiation, bladder increased wall thickness or the presence of ureterocele (4, 10, 11). Timing of the first post-natal US is important, as there is an increased risk of underestimating the severity of hydronephrosis during the first 2 days after birth. This is in part because of the physiological newborn dehydration. The first post-natal US examination should therefore normally not be performed within the first 48 h after birth. A sooner US is only warranted in selected cases (e.g., oligohydramnios, suspicion of urethral obstruction, bilateral high-grade dilation).

The pathologies most frequently discovered by post-natal screening are uretero-pelvic junction stenosis (UPJS), primary vesicoureteral reflux (VUR), megaureter, duplex kidney, and posterior urethral valves (PUV), with UPJS and primary VUR accounting for most of the cases (1, 7, 13, 14).

## Uretero-Pelvic Junction Stenosis (UPJS)

**Figure 2:**
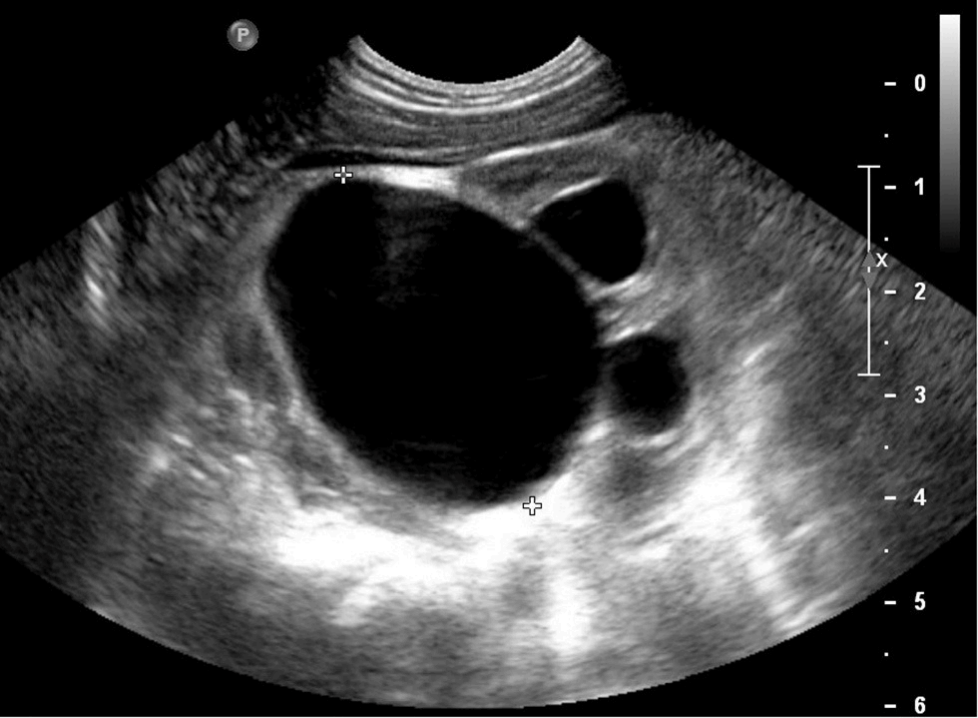
US scan of a severe dilatation in uretero-pelvic junction stenosis.

UPJS occurs in 5–20% of children with antenatally diagnosed renal pelvis dilation (7) and is caused by intrinsic stenosis/valves, peripelvic fibrosis, or crossing vessels at the level of junction between the pelvis and the ureter (15). It is suspected on the observation at US of a dilated renal pelvis (often >15 mm) and calyces in the absence of any dilatation of ureter or bladder. In severe cases, a perirenal urinoma may be seen (3) (Figure 2).

The post-natal management of children with antenatally detected UPJS remains controversial (16, 17). The essential question is whether the child should be operated or managed conservatively. However, the criteria for surgery still vary from center to center and even within the same department (18).

In 2016, the Cochrane review by Weitz et al. (19) aimed at evaluating the effects of surgical vs. non-surgical management in newborns and children < 2 years of age with unilateral UPJS. Unfortunately, the sample size was too small and the follow-up time too short to deliver conclusive results as to which group did better longer term, had fewer complications, and had better quality of life. One year later, Weitz published a systematic survey (20) on more than 1000 patients from mostly observational studies, with the aim of assessing the effect of the non-surgical management of unilateral UPJS. The review showed that the outcome of about 80% of the cases is toward an improved drainage pattern while about 20% of these patients are at risk of split renal deterioration, and 30% will eventually be operated (20). This time the results were biased by the heterogeneity of the included studies and again were unable to resolve the ongoing controversy. Randomized controlled trials with sufficient statistical power and an adequate follow-up period would be needed to define the optimal management.

One of the challenges is probably due to the fact that children with UJPS are often asymptomatic and the criteria for surgery are not clinically-based but mainly related to imaging and isotopic parameters, as the severity of hydronephrosis on US, the level of differential renal function (DRF) and the quality of renal drainage on renogram (17, 20, 21). Moreover, the Pediatric Committee of the European Association of Nuclear Medicine guidelines (22), have underlined the various potential pitfalls in the acquisition, processing and interpretation of isotopic examinations.

Although the indication for pyeloplasty was formerly mainly based on poor drainage (23, 24), physicians are nowadays more hesitant to solely rely on this parameter (25, 26). In fact, recent studies have shown that the transit limited to the cortical area is potentially a good predictive factor for the risk of deterioration in case of conservative treatment (27, 28). The renal transit limited to the cortical area is the passage of the tracer from the outer cortex to the inner structures, as the medulla and collecting system. In physiological situations, the cortical transit is rapid, and a fairly homogeneous kidney filling is observed in approximately 2 min. In the cases of delayed cortical transit, the tracer is retained in the outer cortical rim and the remaining part of the kidney remains hypoactive for several min. A severely delayed cortical transit indicates a kidney at risk of further deterioration if not operated (21).

According to the existing findings, a sensible clinical attitude can be summarized as follows (Figure 1):

- In unilateral UPJS, a conservative approach seems reasonable in the large majority of cases, even in those with severe hydronephrosis on US.
- A rapid renographic control should be planned in the event of a significant increase of the pelvic diameter on US.
- Early surgical intervention is recommended in the cases of deteriorating split renal function and/or delayed cortical transit.

## Primary Vesicoureteral Reflux (VUR)

### Short Term Outcome

**Figure 3:**
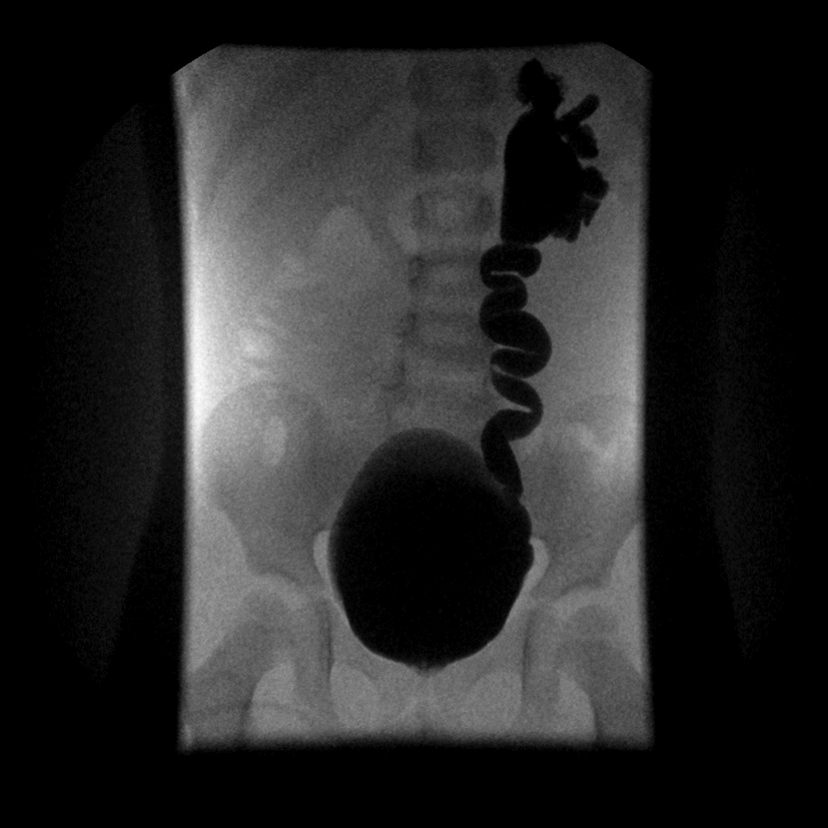
VCUG image of primary vesicoureteral reflux grade V.

VUR is defined as the retrograde flow of urine from the bladder upward within upper urinary tract. The grading system for VUR includes grade I to V which correspond to increasingly severe VUR (29) (Figure 3). VUR is a fairly frequent phenomenon that can be associated with fetal renal pelvis dilatation and in extreme cases with congenital renal dysplasia. Fetal renal pelvis dilation can be an indicator of VUR in 11% (7) to 30% (30) of cases with the lower figure being probably more realistic. It is sometimes suspected *in utero* in cases where intermittent renal collecting system dilatation is seen during real-time scanning (31). The importance of diagnosing neonatal VUR was justified by the perceived risk of pyelonephritis and renal scarring and, ultimately, later in life to hypertension and end-stage renal disease (ESRD) (32). Therefore, previous guidelines suggested to actively search for a VUR in children with antenatally diagnosed dilatation. However, current evidence suggests that only patients with high-grade disease (IV and V) are at real risk for renal dysplasia, serious adverse outcome and delayed resolution (33–35). A large and prospective study found that VUR related to fetal renal pelvis dilation was of low-grade in 74% of cases, with a 2-year spontaneous resolution rate higher than 90% (34). According to these findings, while high grade VUR should not be missed, low-grade reflux is not necessarily clinically significant and there is no real need to routinely search for it, especially in infants with post-natal normal renal ultrasounds (7, 11, 36) (Figure 1). On the contrary, a significant renal pelvis dilatation (≥10 mm), megaureter, and/or the presence of cortical abnormalities on US, warrant the use of voiding cystourethrography (VCUG) for detecting VUR (7, 36).

As to the management of VUR, the role of the continuous antimicrobial prophylaxis (CAP) in the prevention of UTI in children with VUR has been investigated by several prospective studies within the last 10 years. Unfortunately, the inclusion criteria of these studies were very heterogeneous in terms of patient's age and sex, sample size, grades of VUR included and study design. The most reliable studies, the two placebo-controlled, double blind, and largest trials, the PRIVENT (37) and the RIVUR trial (38) showed that prophylaxis is effective in preventing UTI. On the other side, a recent systematic review and meta-analysis by Hewitt et al. (39), which explored the influence of CAP on the risk of renal scarring, found no significant benefit even in children with VUR.

Eventually, the final choice of VUR management (close monitoring, circumcision in boys, antibiotic prophylaxis or surgical treatment by endoscopic injection of bulking agents or ureteral reimplantation) depends on several factors such as the grade of VUR, the clinical course in terms of UTI recurrence, the presence of renal scars, ipsilateral renal function, bilaterality, bladder function, associated anomalies of the urinary tract, age and parental preference (36).

### Long Term Outcome

The traditional assumption that a child with VUR presents an important risk of UTI and renal scarring leading on the long run to adverse outcomes such as hypertension and end-stage renal disease (ESRD) seems to be outdated. The natural course of VUR is heterogeneous and extremely variable. The long-term prognosis for most children with VUR is excellent with a high percentage of spontaneous VUR resolution during childhood (34, 35). According to the RIVUR study (38), <15% of children with VUR show progression of renal scarring, and this independently on the use of prophylaxis. This population at risk of serious complications, is mostly constituted by the small group of children with pre-existing renal damage (40). Congenital hydronephrosis due to high-grade VUR for example is often accompanied by a variable degree of renal dysplasia (41). The most severely affected infants progress to ESRD within the first years of life, while those with less severe congenital malformations typically undergo a transient period of stable renal function until puberty, and often reach ESRD during adolescence. According to these findings, a sensible clinical attitude can be summarized as follow (Figure 1):

- In post-natal care, the use of VCUG is recommended with US findings of:
- Pelvis dilatation ≥10 mm
- Signs of hypodysplasia
- Ureteral dilatation
- Abnormal bladder
- In patients with lower grade VUR (grade I-II) and no symptoms, close surveillance without antibiotic prophylaxis is recommended
- In children presenting with dilating RVU (grade III-V), CAP is the preferred option for initial therapy. Circumcision should be considered in boys with breakthrough UTI. In patients with dilating VUR or abnormal renal parenchyma, surgical repair is a sensible alternative.
- In patients with high risk of renal damage as in case of febrile UTI recurrence in the context of high-grade RVU, bilaterality and/or cortical abnormalities, a surgical approach is often proposed.
- Children with bilateral high-grade VUR and renal damage already existing at diagnosis need a long-term follow-up.

## Megaureter

*In utero*, megaureter appears as a serpentine fluid-filled structure with or without dilatation of the renal pelvis and calices (11). Ureteral dilatation may be due to primary megaureter (pMU) which is the obstruction at the level of the junction between ureter and bladder. Secondary megaureter is associated to an underlying condition (e.g., high-grade reflux, neurogenic bladder, or posterior urethral valves), and the differential diagnosis relies on VCUG.

Prognosis of pMU is generally good with a high rate of spontaneous resolution after 1–3 years. However, the risk of pyelonephritis and the need for continuous antibacterial prophylaxis in asymptomatic newborns with pMU remain a subject of controversy (42). Some authors recommend CAP at least during the first 6 months of life (43), although for others, in the absence of recurrent UTIs and/or VUR CAP is not considered as mandatory (3). A recent review on a 23-year period-observation of pMU at a German tertiary academic center, showed a steady decline of surgical interventions during the last three decades due to the positive outcomes of conservative therapy in the majority of the children (43). However, in cases of severe hydronephrosis, or a retrovesical ureteral diameter >10 mm, the condition may take time to resolve (11). According to the British Association of Pediatric Urologists (BAPU), surgical intervention is indicated in the presence of symptoms as recurrent febrile UTIs, impaired renal function associated with massive or progressive hydronephrosis, or a drop in differential function on serial renograms (44). In these cases, the BAPU recommends a ureteral reimplantation in patients over 1 year of age even though the procedure may be challenging in small children (44).

According to these findings, a sensible clinical attitude can be summarized as follow:

- In children with asymptomatic pMU, close surveillance is recommended.
- In the absence of recurrent UTIs, antibiotic prophylaxis is not mandatory.
- Surgical intervention is often required in case of recurrent febrile UTIs and/or deteriorating split renal function on serial renograms.

## Duplex Kidney

**Figure 4:**
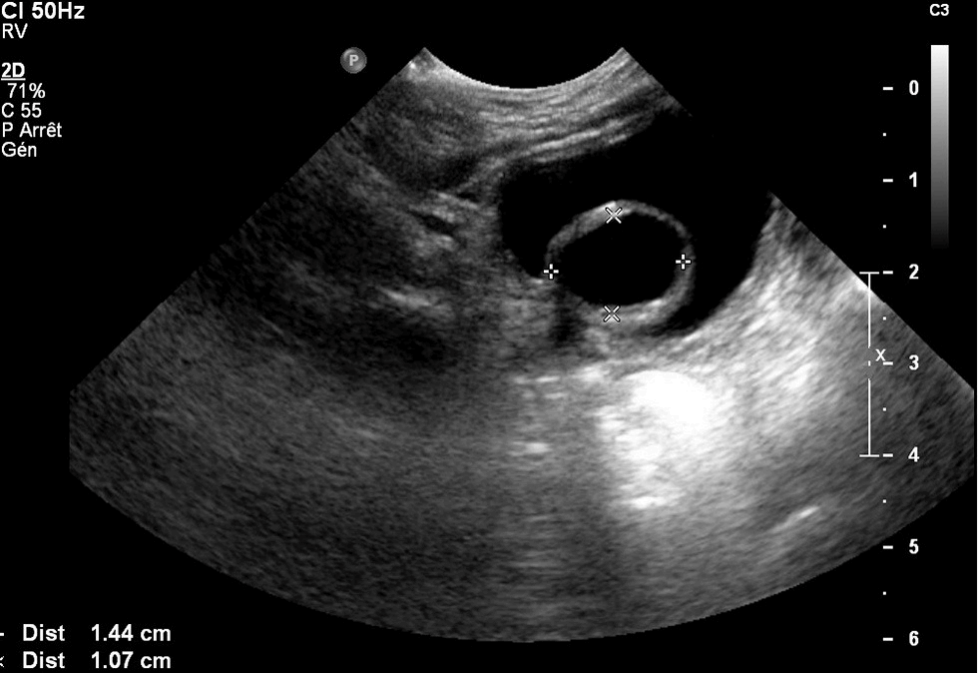
US scan of obstructive ureterocele in a duplex system.

Duplication of the renal collecting system is a congenital defect that involves one kidney drained by two ureters that may be completely or partially separated (45). Duplex kidney should be considered as a normal variant when the cavities are non-dilated and renal impairment is not present (7). However, it can also be associated with the presence of VUR and/or obstruction. Fetal urinary tract dilatations are associated to the presence of complicated renal duplications in <5% of cases (7). In duplex kidney, VUR classically involves only the lower pole ureter and tends to be of higher grade as compared to a single system reflux (45, 46). Obstruction of the upper pole may be due to ureteroceles in 80% of cases, although it may also occur secondary to an ectopic insertion or an isolated pMU (45) (Figure 4). *In utero*, duplex kidneys may be seen as two non-communicating renal pelves, cystic structures within one pole, and a sac-like pouch in the bladder, representing ureterocele (47).

The classical investigations performed after birth are US and VCUG (48). Most authors agree that the surgical approach to complicated duplex systems is largely predicted by the clinical evolution and the presence or absence of function in the affected renal moiety (48). According to these findings, a sensible clinical attitude can be summarized as follow:

- In children with dilated duplex system, US and VCUG are recommended after birth in order to search for VUR and to detect the presence of ureterocele.
- In complex cases, isotopic studies are recommended in order to evaluate renal function in the dilated renal moiety.
- Surgical approach should be proposed in cases of obstructive ureterocele (ureterocele puncture), and/or absence of function in the diseased moiety (heminephrectomy).

## Posterior Urethral Valves

### Short Term Outcome

**Figure 5:**
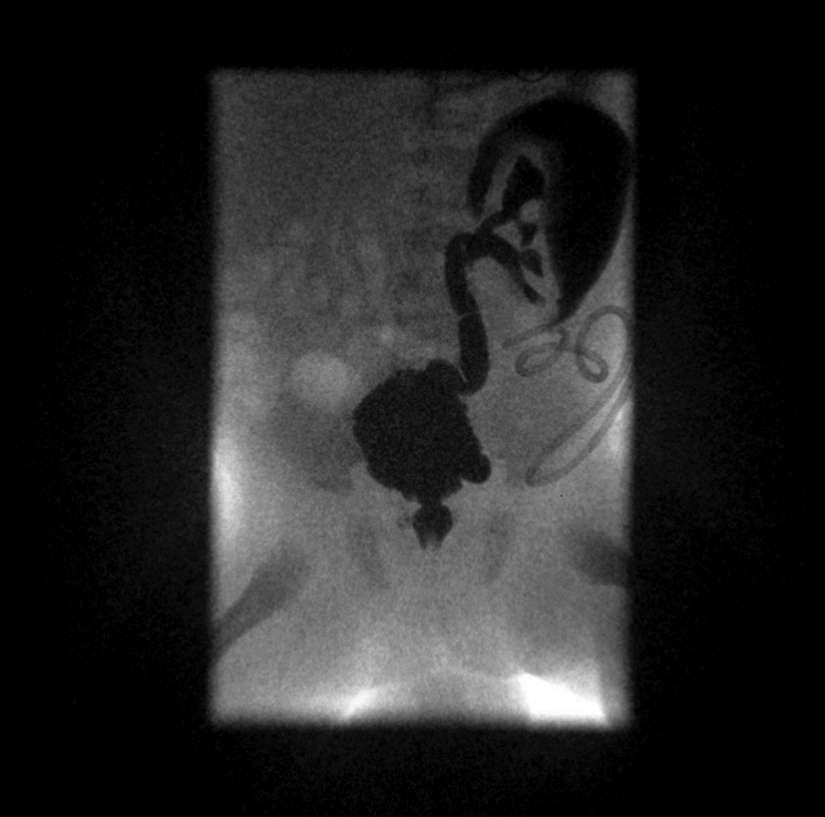
High-grade RVU and megabladder with diverticula in a child with posterior urethral valves.

Posterior Urethral Valves (PUV) are tissue leaflets fanning distally from the prostatic urethra to the external urinary sphincter. When suspected in the first trimester of pregnancy, PUV carrie a very poor prognosis (49). In the second trimester, PUV represent the most common cause of lower urinary tract obstruction in boys, affecting 1 in 4,000–8,000 infants. *In utero*, PUV should be suspected in the following situations: failure of the bladder to empty, presence of abnormal kidneys and oligohydramnios. Sometimes a megabladder with a thickened wall may be seen, and the dilated posterior urethra may take the aspect of a keyhole. In extreme cases *in utero*, kidney or bladder rupture may be observed with extravasation of urine resulting in urinary ascites (50) (Figure 5).

The prognosis is often relatively easy to predict in severe cases, such as presentation before 24 weeks, oligohydramnios, and increased cortical echogenicity (51). In those situations perinatal death will occur secondary to pulmonary hypoplasia and renal failure (52). In partial obstruction, the outcome is less predictable, and late morbidity most commonly takes the form of end-stage renal failure, which affects 15 to 30% of children after birth (53). Once PUV are prenatally suspected, management warrants the involvement of a multi-disciplinary team in a fetal and pediatric surgery reference center. Various options might be discussed according to the severity of presentation, including termination of pregnancy, *in utero* therapy or follow-up with planned post-natal management.

For decades, a variety of *in utero* therapeutic approaches to relieve obstructing posterior urethral valves have been tried: open surgical technique of fetal vesicostomy (54), direct endoscopic valves resection (55, 56), and vesicoamniotic shunting (57).

Vesicoamniotic shunting performed under US guidance using a pigtail shunt, seems to be the preferred technique for bladder drainage (58, 59). In 2013, Morris et al. (60) presented the results of the PLUTO (Percutaneous vesicoamniotic shunting in Lower Urinary Tract Obstruction) study. In this trial, fetuses with fetal PUV were randomly assigned to either vesicoamniotic shunting or conservative management. PLUTO's results have shown that post-natal survival was three-times higher in the fetuses receiving vesicoamniotic shunting.

However, only two out of seven shunted survivors had normal renal function at 1 year of age.

These results suggest that the chance of newborn babies to survive with normal renal function is very low irrespective of whether or not vesicoamniotic shunting is performed (61).

In conclusion, the experience of the intrauterine shunting techniques as currently practiced suggests that post-natal survival may be improved. However, little if any improvement of post-natal renal function can be achieved. At present time, the best that modern medicine has to offer to these children in order to improve their long-term health is close urological care following a full gestation delivery with the aim to maximize the bladder and renal function.

Long term outcome PUV are the most frequent cause of chronic renal disease in boys and account for about 17% of children with ESRD (62). The incidence of renal and bladder dysfunction in PUV patients varies widely, as the clinical spectrum range from early presentation with severe renal dysplasia to late presentation with mild lower urinary tract symptoms and recurrent UTI. In a systematic review (63) on the outcomes of PUV on nearly 1500 patients, the percentage of CKD and ESRD was of more than 20 and 10%, respectively, while urodynamic bladder dysfunction was seen in more than half of the patients after endoscopic treatment of PUV, and nearly one in five cases was reported to suffer from urinary incontinence. In general, the patients with the most severe sequelae of PUV and the higher risks of chronic kidney disease and ESRD are diagnosed early in childhood and are at the most somber side of the PUV spectrum. In a retrospective study on more than 100 patients with PUV, antenatal diagnosis, prematurity, abnormal renal cortex, and loss of cortico-medullary differentiation on initial US and elevated plasma creatinine at 1 year of age were the factors associated with the higher risk of CKD and ESRD (64).

According to these results, a sensible clinical attitude can be summarized as follow:

- Antenatal treatment of PUV is still experimental; therefore treatment should be started at birth and no sooner.
- All cases antenatally suspected with a PUV should be referred to a neonatal intensive care unit with urological expertise.
- In all children suspected of PUV, catheter drainage of the bladder should be performed at birth, with a close monitoring of serum electrolytes, renal function and antibiotics to prevent UTI.
- In all children suspected of PUV, VCUG should be performed as soon as possible as it is the only direct investigation to diagnose valves; a voiding view of the urethra with the catheter removed is crucial in order to make a complete evaluation of the urethra.
- Endoscopic valves ablation should be planned when the child is medically stable.
- If catheter drainage improves hydronephrosis and plasma creatinine, then valve ablation is all that is necessary.
- If catheter drainage improves hydronephrosis but renal function deteriorates, this points to dysplastic kidneys.
- If with catheter drainage the hydronephrosis and renal function deteriorates, an upper tract diversion should be discussed.
- If the newborn is too small (under 2 Kg), the urethra might not allow safe resectoscope introduction. In these cases, vesicotomy can be performed in order to alleviate the obstruction until the child is big enough for definitive treatment.
- After valve ablation a close follow-up is recommended to ensure proper bladder function.
- Renal function should be assessed and followed on the long run.

## Author Contributions

We certify that all authors listed on the manuscript have participated in the present work. BC: drafting the work and revising it; MG: providing images, drafting the work and revising it; KK: drafting the work and revising it; KI: Drafting the work and revising it.

### Conflict of Interest Statement

The authors declare that the research was conducted in the absence of any commercial or financial relationships that could be construed as a potential conflict of interest.
